# Pulmonary Bacteriophage Therapy on *Pseudomonas aeruginosa* Cystic Fibrosis Strains: First Steps Towards Treatment and Prevention

**DOI:** 10.1371/journal.pone.0016963

**Published:** 2011-02-15

**Authors:** Eric Morello, Emilie Saussereau, Damien Maura, Michel Huerre, Lhousseine Touqui, Laurent Debarbieux

**Affiliations:** 1 Molecular Biology of the Gene in Extremophiles Unit, Department of Microbiology, Institut Pasteur, Paris, France; 2 Histotechnology and Pathology Unit, Department of Infection and Epidemiology, Institut Pasteur, Paris, France; 3 Innate Host Defense and Inflammation Unit, Department of Infection and Epidemiology, Institut Pasteur, Paris, France; 4 INSERM, U874, Paris, France; Cairo University, Egypt

## Abstract

Multidrug-resistant bacteria are the cause of an increasing number of deadly pulmonary infections. Because there is currently a paucity of novel antibiotics, phage therapy—the use of specific viruses that infect bacteria—is now more frequently being considered as a potential treatment for bacterial infections. Using a mouse lung-infection model caused by a multidrug resistant *Pseudomonas aeruginosa* mucoid strain isolated from a cystic fibrosis patient, we evaluated bacteriophage treatments. New bacteriophages were isolated from environmental samples and characterized. Bacteria and bacteriophages were applied intranasally to the immunocompetent mice. Survival was monitored and bronchoalveolar fluids were analysed. Quantification of bacteria, bacteriophages, pro-inflammatory and cytotoxicity markers, as well as histology and immunohistochemistry analyses were performed. A curative treatment (one single dose) administrated 2 h after the onset of the infection allowed over 95% survival. A four-day preventive treatment (one single dose) resulted in a 100% survival. All of the parameters measured correlated with the efficacy of both curative and preventive bacteriophage treatments. We also showed that *in vitro* optimization of a bacteriophage towards a clinical strain improved both its efficacy on *in vivo* treatments and its host range on a panel of 20 *P. aeruginosa* cystic fibrosis strains. This work provides an incentive to develop clinical studies on pulmonary bacteriophage therapy to combat multidrug-resistant lung infections.

## Introduction

*Pseudomonas aeruginosa* is the second most common pathogen responsible for hospital-acquired bacterial pneumonia as well as ventilator-associated pneumonia, and the first causative agent of morbidity and mortality in cystic fibrosis (CF) patients [1], [2]. Although antibiotics are still an effective means of treating bacterial lung infections, the alarming rise of multidrug-resistant bacteria in hospitals has highlighted the need for new therapies [3], [4], [5]. Bacteriophages —viruses infecting bacteria— have been proposed to treat human bacterial infections since their discovery in the early 20th century [6], [7]. However, after a short period of development, the advent of antibiotics led to this therapeutic approach being abandoned, except in Eastern Europe where bacteriophages are still used today to treat patients [8], [9], [10]. During the past 20 years, studies in animal models have demonstrated the potential of bacteriophages [11], [12], [13], [14], [15]. Recently the first phase II clinical trial performed under European regulations on bacteriophage treatments of chronic otitis was published, and demonstrated the interest of using bacteriophages on multidrug resistant infections [16]. The effects of bacteriophage therapy on lung infections has only very recently been addressed in animal models [14], [17]. On the one hand, a proof of concept with a bioluminescent strain of *P. aeruginosa* showed that bacteriophages administrated intranasally had a rapid efficacy with respect to preventing and curing deadly lung infections [14]. On the other hand, a clinical strain of *Burkholderia cenocepaci*a isolated from a CF patient was used to show that the intraperitoneal administration of bacteriophages was more effective than intranasal applications in a non-deadly infectious model [17]. Here we report an in-depth evaluation of the efficacy of curative and preventive bacteriophage treatments of lung infections using a multidrug resistant mucoid *P. aeruginosa* strain isolated from a CF patient of Grenoble hospital, France [18], [19]. For this study we specifically optimized the virulence of a bacteriophage of our collection towards this clinical strain and studied its efficacy both *in vitro* and *in vivo*.

## Results

### Characterization of a lung infection by a *P. aeruginosa* strain isolated from a cystic fibrosis patient

**Figure 1:**
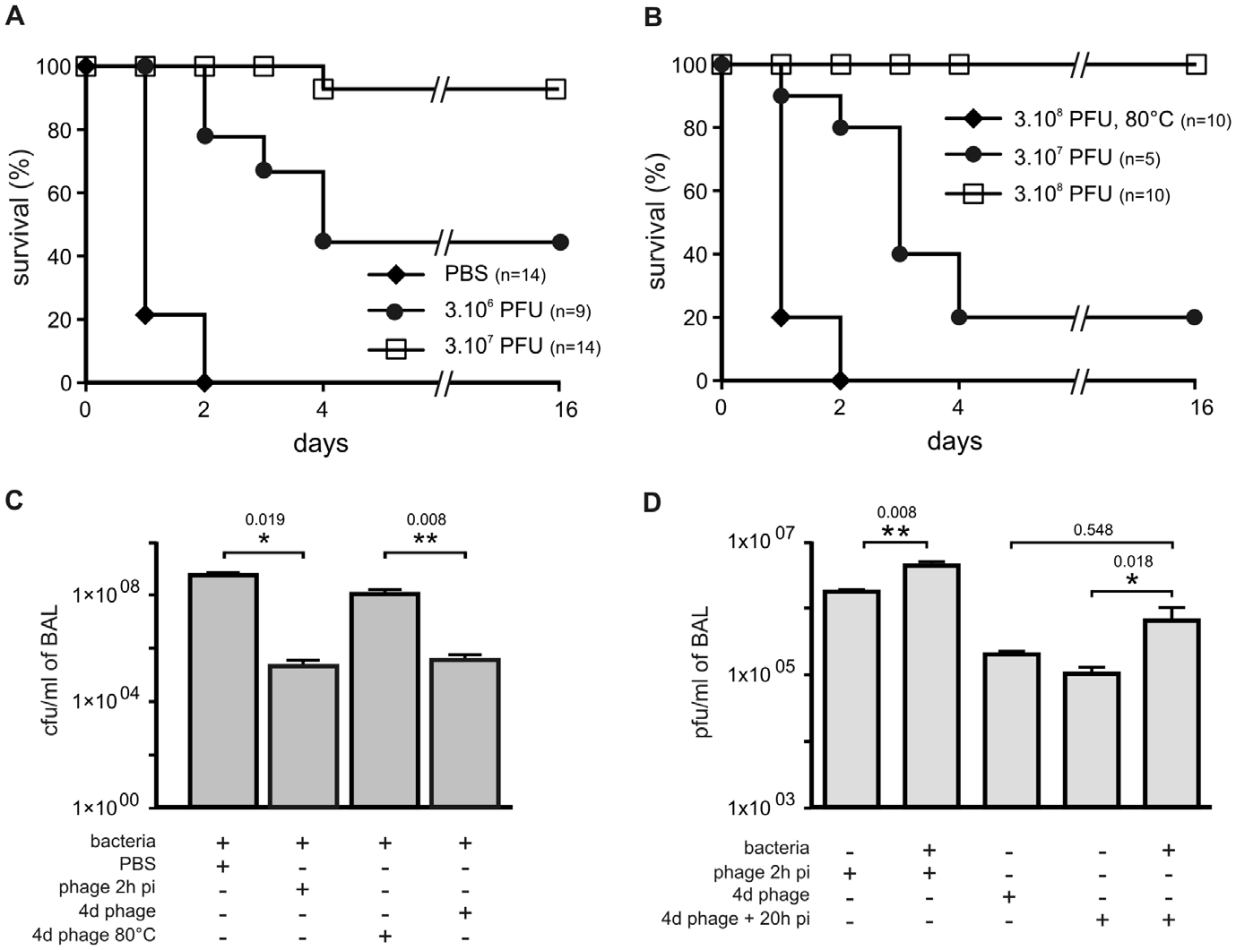
Bacteriophage P3-CHA cure and prevent lung infections caused by a clinical *P. aeruginosa* strain. (**A–B**) Survival curves of mice infected with the CHA strain and treated or pre-treated with P3-CHA bacteriophage. (**A**) PBS (♦), 3×10^6^ (•) and 3×10^7^ (□) pfu of bacteriophage were given intranasally 2 h after bacteria (3×10^6^ cfu) were administered. This curative treatment appears to be dose dependent (P<0.0001 for both bacteriophage doses compared to PBS and P<0.01 between 3×10^6^ and 3×10^7^ bacteriophage doses). (**B**) Four days before infection with 3×10^6^ bacteria, mice were given either 3×10^7^ (•), or 3×10^8^ (□) pfu of P3-CHA or 3×10^8^ pfu of heat-inactivated P3-CHA solution (♦). These survival curves indicate that the preventative treatment is dose dependent (P<0.0005 and P<0.0001 for 3×10^7^ and 3×10^8^ bacteriophage doses respectively compared to heat-inactivated bacteriophage solution and P<0.0005 between 3×10^7^ and 3×10^8^ bacteriophage doses). (**C–D**) 20 h after infection with strain CHA, mice were euthanized and BAL fluids were assayed for bacteria (**C**) and bacteriophages (**D**). In the curative treatment protocols, mice were treated with PBS or bacteriophage 2 h post infection (phage 2 h pi). In the preventative treatment protocols, mice were intranasally administered bacteriophage solution (4d phage) or heat-inactivated bacteriophage solution (4d phage 80°C) four days before infection. (**C**) Bacterial counts were significantly lower in the BAL fluids from mice that had received either curative or preventative bacteriophage treatment than the respective control treatment (* P<0.05, and ** P<0.01). (**D**) Bacteriophage counts were significantly lower in the BAL fluids from mice that had received bacteriophage treatment than the non-infected animals (** P<0.01) or the non-infected animals pre-treated four days earlier with the bacteriophage P3-CHA (* P<0.05).

**Figure 2:**
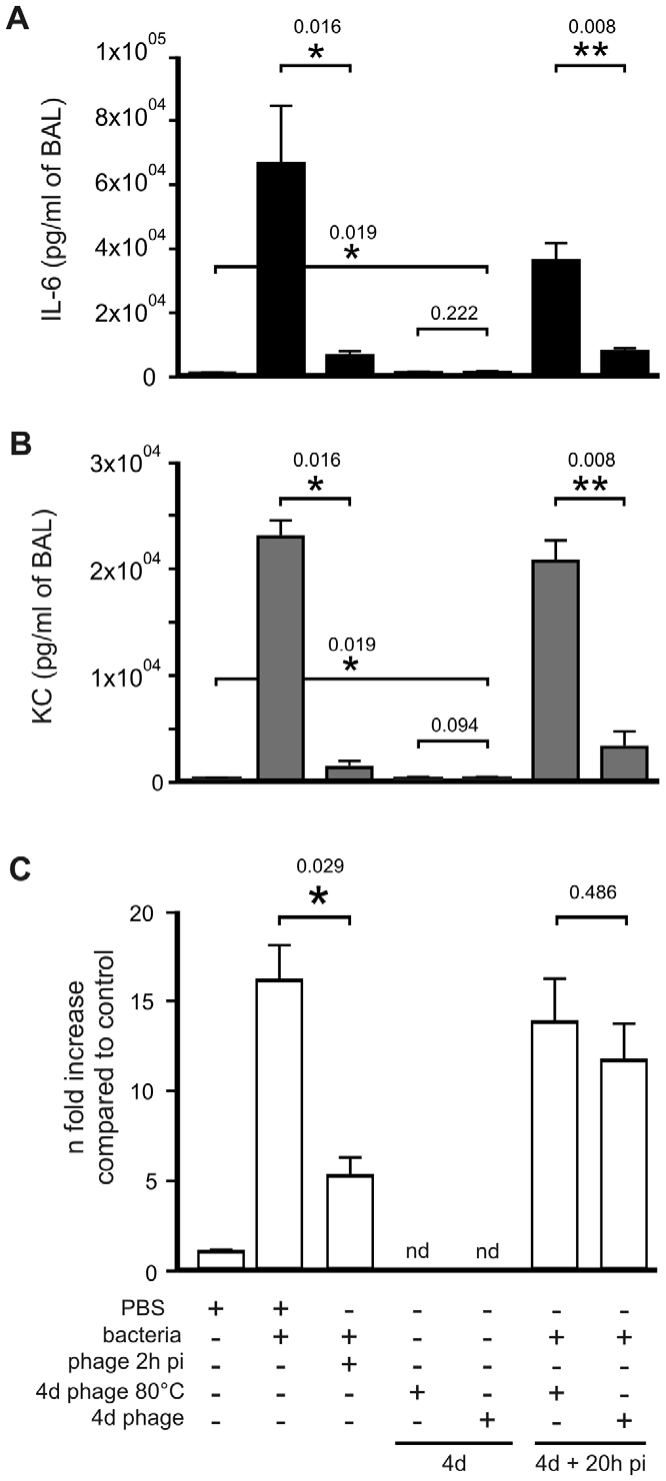
Inflammatory and cytotoxicity analyses during bacteriophage P3-CHA treatments. (**A–C**) 20 h after infection with strain CHA, mice were euthanized and BAL fluids were assayed for cytokines (**A** and **B**) and LDH (**C**). In the curative treatment protocols, mice were treated with PBS or bacteriophage (phage 2 h pi) 2 h post infection. In the preventative treatment protocols, mice were intranasally administered bacteriophage solution (4d phage) or heat-inactivated bacteriophage solution (4d phage 80°C) four days before infection. IL-6 (**A**), KC (**B**) concentrations in BAL fluids from mice that had received either curative or preventative bacteriophage treatments were significantly lower than the values for their respective controls untreated animals ((**A**) * P<0.05, (**B**) * P<0.05) and animals pre-treated with heat-inactivated bacteriophage solution ((**A**) ** P<0.01, (**B**) ** P<0.01). LDH (**C**) levels were significantly reduced only in mice that had received a curative treatment (* P<0.05). n = 5 to 7 for each condition (**A–C**). nd, not determined.

**Figure 3:**
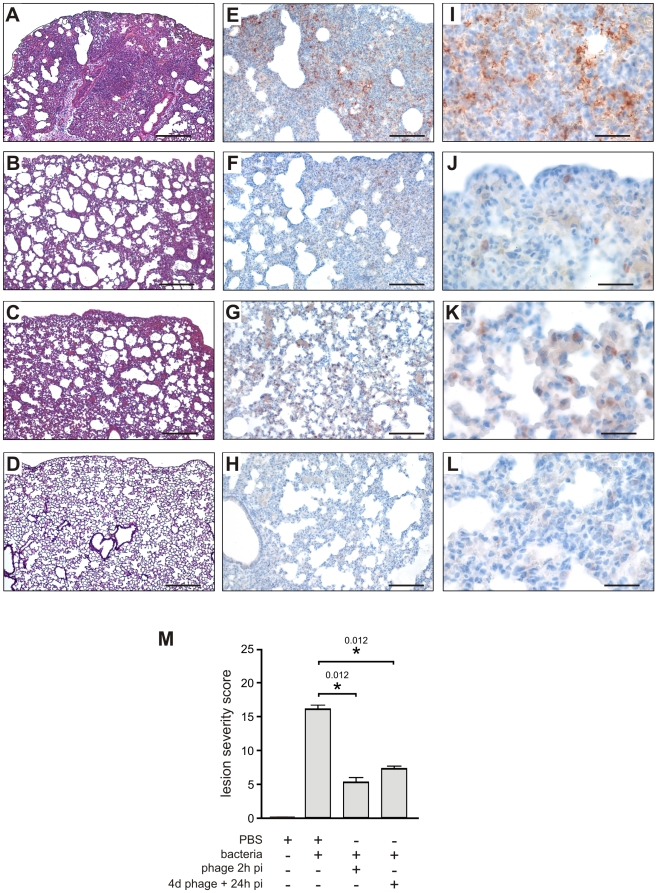
Histological and immunohistochemical analyses of lung sections from mice treated or pre-treated with bacteriophage P3-CHA. (**A–D**; scale bar 200 µm) Thin sections of lungs obtained after 20 h from, (**A**) mice infected with the CHA strain, (**B**) infected mice treated with bacteriophage P3-CHA, (**C**) mice pre-treated with bacteriophage P3-CHA four days before infection, and (**D**) uninfected mice were stained with hematoxylin-eosin and Gram. Histological analyses included the determination of a lesion severity score (see materials and methods) to allow comparison (**M**). This score was significantly lower for mice given curative or preventive bacteriophage treatments than for untreated mice (* P<0.05). (**E–L**) Immunohistochemistry was performed with anti-*Pseudomonas* antibodies on sections obtained from the same samples as above (**E–H**; scale bar 100 µm and **I–L**; scale bar 50 µm). In lung sections from infected mice, bacteria are both intracellular and extracellular (**E** and **I**) whereas no signal can be seen in lung sections from uninfected mice (**H** and **L**). The signal was lower following either curative (**F** and **J**) or preventive (**G** and **K**) bacteriophage treatments and bacteria were only observed in macrophage cells.

To investigate bacteriophage treatments on a mouse lung-infection caused by a clinical *P. aeruginosa* strain named CHA [18], [19], we first established the conditions in which an intranasal administration of this strain was lethal. We found that inoculation of 3×10^6^ bacteria was sufficient to induce death in 100% of animals within 2 days (Figure 1A). Progress of the infection was assessed by quantification of bacteria, inflammatory markers, and cytotoxicity levels at 20 h post-infection (Figures 1 and 2). The number of bacteria in the lungs had increased at least by two orders of magnitude compared with the initial infectious dose (over 4×10^8^ cfu were found in the broncho-alveolar lavages [BAL] form each infected mouse compared with the infectious dose of 3×10^6^ cfu; Figure 1C). The levels of two pro-inflammatory markers (cytokines IL-6 and KC) as well as lactate dehydrogenase (LDH) which reflects organ toxicity, were highly elevated (Figure 2). Histological analysis, also at 20 h post-infection, revealed severe lesions consistent with acute pneumopathy, combined with focal and diffuse alveolitis and bronchitis, consolidation and necrosis (Figure 3A compared to Figure 3D from uninfected animals). Disease severity was scored at 16 out of 25 (Figure 3M). These findings confirmed that the clinical *P. aeruginosa* strain CHA is able rapidly to infect lungs and induce severe damage leading to death in these mice.

### Bacteriophage selection and characterization

**Figure 4:**
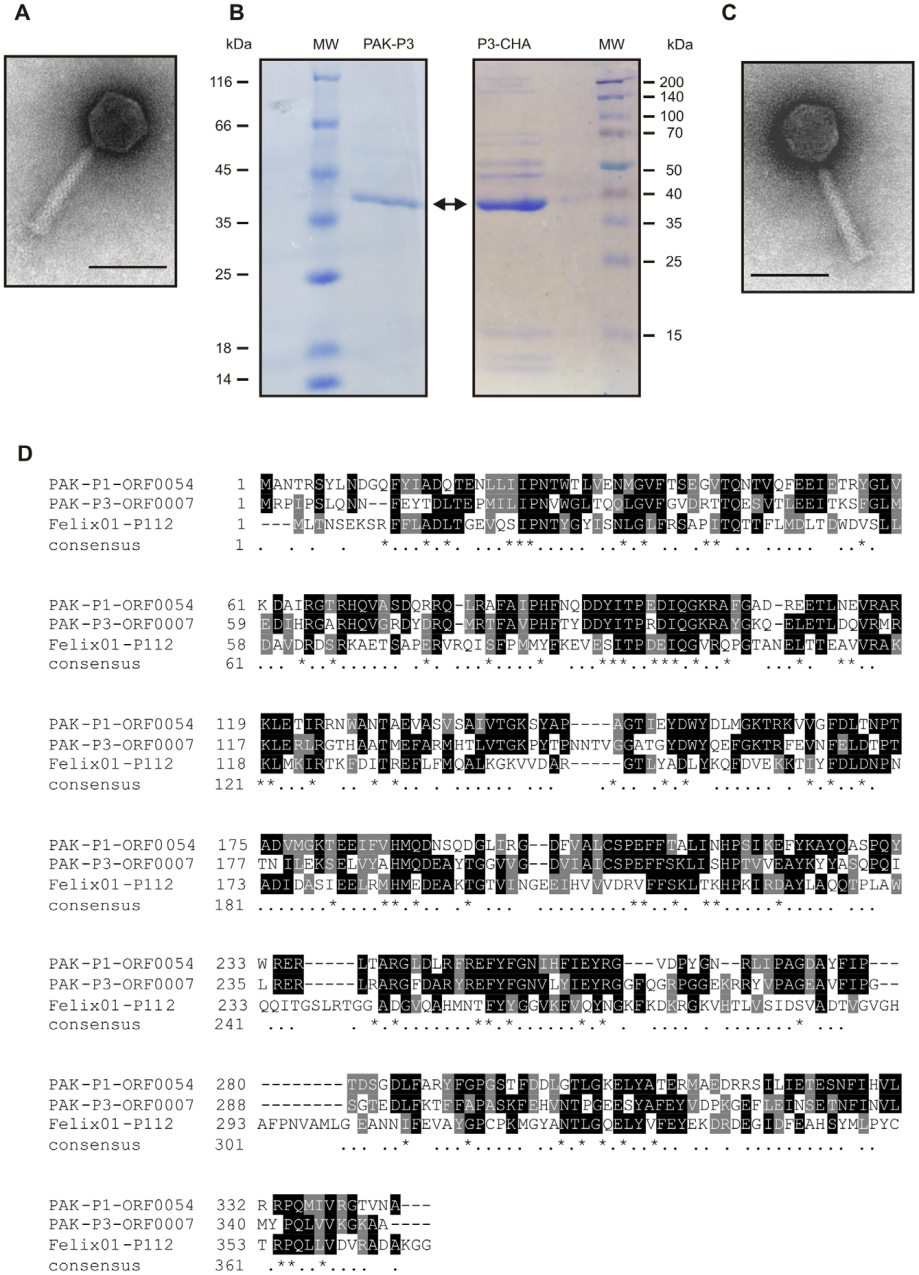
Characterization of PAK-P3 and P3-CHA bacteriophages. (**A**, **C**) Electron micrographs of PAK-P3 (**A**) and P3-CHA (**C**). Scale bar: 100 nm. (**B**) SDS-PAGE of PAK-P3 and P3-CHA proteins; only the most abundant proteins give visible signals (MW: molecular weight markers, the arrow points to the major capsid proteins). (**D**) Clustal alignment of the three major capsid proteins of PAK-P1, PAK-P3 and P3-CHA bacteriophages with their closest homologs in the database with known function (the major capsid protein of Felix 01 bacteriophage; NP_944891). Major capsid proteins of PAK-P3 and P3-CHA are 100% identical.

We screened our collection of natural *P. aeruginosa* bacteriophages (initially isolated from the environment using the *P. aeruginosa* strain PAK [14]) and found that the PAK-P3 bacteriophage was able to establish a moderate infection in strain CHA (plating efficiency of 10% compared with 100% on the PAK strain). After five consecutive passages in liquid culture with the CHA strain we obtained a bacteriophage stock that showed the same plating efficiency on both CHA and PAK strains. Subsequently, four steps of plaque purification were performed to obtain a preparation of a single isolated phage that we named P3-CHA (see Materials and Methods). Electron microscopy showed that both bacteriophages (PAK-P3 and P3-CHA) belong to the Myoviridae family of bacterial viruses (Figure 4). Their genomes were sequenced (Genbank accession numbers: HM173082 for PAK-P3, and HM173081 for P3-CHA) and analysed revealing that they are distantly related to the PAK-P1 bacteriophage [14]. Major capsid proteins were identified by mass spectrometry (Figure 4). Putative proteins encoded by their genomes were analysed and no significant similarities to proteins considered to be markers of temperate bacteriophages or toxins were identified (see Materials and Methods). This confirmed that both PAK-P3 (vB_PaeM_PAK_P3) and P3-CHA (vB_PaeM_P3_CHA) are virulent bacteriophages. We estimated the host range of these two bacteriophages with a set of 20 clinical CF strains (Table 1). The P3-CHA bacteriophage was overall more efficient than PAK-P3 on infecting *P. aeruginosa* strains isolated from both primary and chronically infected patients.

**Table 1:** Efficacy of plating of PAK-P3 and P3-CHA bacteriophages on CF *P. aeruginosa* strains.

Efficacy of plating relative to the reference strain, %										
	Strains from patients with primary infections									
	1	2	3	4	5	6	7	8	9	10
PAK-P3	0	0	0	35	6	33	0	66	0	0
P3-CHA	0.01	0.01	0	73	40	100	0.01	100	0.007	0
	Strains from patients with chronic infections									
	1	2	3	4	5	6	7	8	9	10
PAK-P3	0	20	0	0	8	0	0	0	100	0
P3-CHA	0	100	0.07	0	100	0	0	0	100	0

Efficacies of PAK-P3 and P3-CHA bacteriophages on their reference strains, respectively. PAK and CHA strains were fixed at 100%. Results are expressed as relative percentage to these references.

### Curative treatment

We administered two different doses of P3-CHA bacteriophage intranasally (mimicking a nebulisation treatment for humans) to two groups of mice that had received a lethal dose of the CHA strain 2 h earlier and followed their survival (Figure 1A). Both doses of bacteriophage improved survival and the high dose (3×10^8^ pfu) was associated with a greater rate of survival than the low dose (3×10^7^ pfu) throughout the experiment (16 days). Twenty hours after inoculation (*i.e.* 18 h after bacteriophage treatment was given) we quantified bacteria, bacteriophages, cytokines, and LDH, as well as performing histological analyses. In the group treated with the high dose, the number of bacteria was over two orders of magnitude lower than in the untreated group (Figure 1C), and the number of bacteriophages was ten times higher (Figure 1D). Cytokines and LDH (Figure 2) concentrations were markedly lower in the bacteriophage-treated group than in the untreated group. Histological analyses confirmed that lung damage in the treated group was less severe than in the untreated animals. In the bacteriophage-treated mice, moderate or mild lesions of alveolitis and bronchitis were observed without necrosis or consolidation; in a blind protocol, the severity of these histological lesions was scored at 5 out of 25 (Figure 3B and M). Immunohistochemistry, using a polyclonal anti-*Pseudomonas* antibody, detected only a few bacteria (entire cells or debris) in bacteriophage-treated animals, mainly in the cytoplasm of macrophages in the lungs (Figure 3F and J). In contrast, bacteria were detected in macrophages, alveolae, and extracellular spaces of the lungs from untreated animals (Figure 3E and I). No signal was detected in uninfected animals (Figure 3H and L). These observations are consistent with the fact that bacteriophages target only extracellular bacteria and also show the role of phagocytosis in bacterial removal. These experiments indicate that a curative bacteriophage treatment acts cooperatively with the immune response to eliminate acute lung infection caused by a CF strain of *P. aeruginosa*.

**Figure 5:**
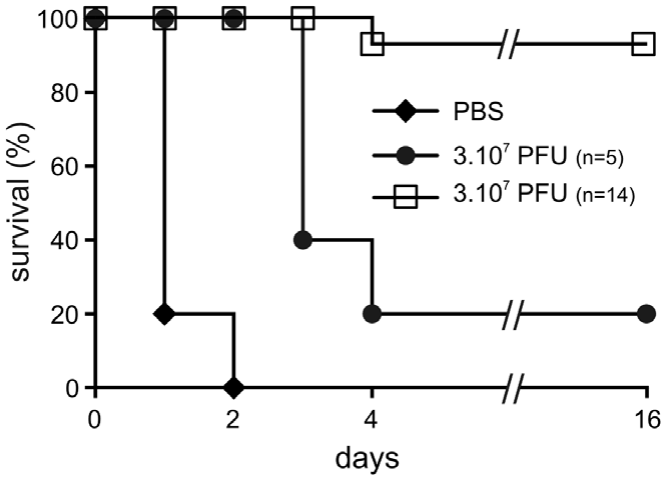
Survival curve of infected mice treated with bacteriophage PAK-P3 compared to P3-CHA. Mice were infected intranasally with 3×10^6^ cfu (CHA strain) and 2 h later were treated with either PBS (♦) or 3×10^7^ pfu of bacteriophage PAK-P3 (•), or 3×10^7^ pfu of bacteriophage P3-CHA (□) also administered intranasally (P<0.005 for both P3-CHA and PAK-P3 bacteriophage doses compared to PBS and P<0.05 between P3-CHA and PAK-P3 bacteriophage doses).

We also used survival-curve analysis to assess the curative efficacy of the PAK-P3 parental bacteriophage (used in the same conditions as P3-CHA, Figure 5). After 8 days, only 20% of treated mice had survived. Thus, the *in vivo* protection in this model correlated with *in vitro* plating efficiency, as previously hypothesised [14]. Moreover the *in vivo* advantage of P3-CHA appears to be only due to two single nucleotide changes in the entire genome, highlighting the plasticity of the bacteriophage genomes to rapidly increase their infectivity to new hosts (see Materials and Methods).

### Preventive treatment

**Figure 6:**
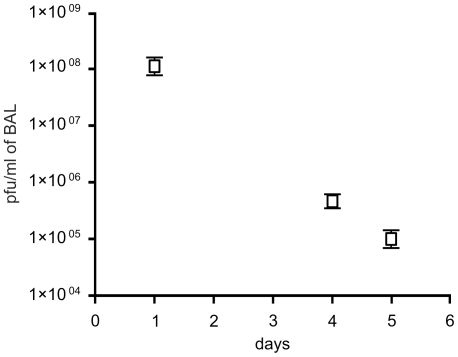
Persistence of P3-CHA bacteriophage in lungs of uninfected mice. Three groups of five mice were given 3×10^8^ pfu of bacteriophage P3-CHA, administered intranasally. The number of bacteriophages in BAL fluids was determined 1, 4 and 5 days post-administration (n = 5).

To determine whether the P3-CHA bacteriophage could be used to prevent an infection by the CHA strain, we prepared an endotoxin-free bacteriophage solution (see Materials and Methods). This reduced the possibility of stimulating an immune response in the host, which could mask the effects of bacteriophage treatment. We first determined the rate of elimination of the P3-CHA bacteriophage from the lungs of uninfected mice and found that its concentration decreased by slightly more than half-log/day (over 500 fold decrease between day 0 and day 4, Figure 6). Only 20% of animals given 3×10^7^ bacteriophages four days prior to infection with 3×10^6^ bacteria were protected whereas 100% of the animals given 3×10^8^ bacteriophages were protected (Figure 1B). 100% of the animals pre-treated 4 days before infection with the equivalent of 3×10^8^ pfu of heat-killed P3-CHA solution died within 2 days (Figure 1B), showing that an active bacteriophage is required. Twenty hours after infection of a group of mice pre-treated with P3-CHA bacteriophage (3×10^8^ pfu) four days earlier, animals were euthanized to perform histological analyses and to quantify bacteria, bacteriophages, cytokines, and LDH. Bacterial counts were lower in P3-CHA pre-treated mice than in heat-killed P3-CHA pre-treated mice (Figure 1C). Bacteriophage counts were higher in P3-CHA pre-treated mice than in non-infected P3-CHA pre-treated mice (Figure 1D). Cytokine concentrations were also significantly lower for P3-CHA pre-treated mice, whereas LDH concentrations were similar in both groups (Figure 2). Furthermore the level of both active and heat-inactivated solutions of bacteriophage gave rise to identically low levels of pro-inflammatory markers (Figure 2). Histological analyses showed that lung damage was markedly less severe in P3-CHA pre-treated mice than the controls, with histological lesions being scored at 7 out of 25 (Figure 3C and M). Immuno-histochemistry of lungs from mice that were pre-treated with P3-CHA bacteriophages showed a pattern that was similar to curative treatment. Bacterial antigens were detected only in the cytoplasm of alveolar macrophages (Figure 3G and K). These data confirmed the efficacy of the preventive bacteriophage treatment.

## Discussion

A clinical strain of *P. aeruginosa* isolated from a CF patient was used to evaluate bacteriophage curative and preventive treatments on a lung infection model. Both treatments successfully rescued mice from lethal infections, underlying the potential use of bacteriophages to combat lung pathogens. We also showed that pro-inflammatory and cytotoxicity markers, as well as histology observations, were all concordant with these results for survival.

Moreover, we observed that bacterial debris released during bacteriophage treatments were not pro-inflammatory, as production of cytokines was not strongly stimulated in either the curative or the preventive treatments. A pro-inflammatory response is often used as an argument against phage therapy. Four days after administration of the preventative treatment that consisted of intranasal bacteriophage solution only, the lung concentrations of IL-6 and KC were low (Figure 2A and B). The main cause of bacterial destruction can therefore be attributed to the bacteriophage itself. However, the cytokine concentrations were still significantly higher than those measured 24 h after administration of a PBS control solution, and partial involvement of a weak immune response stimulated by the bacteriophage solution cannot thus be ruled out. Indeed, the quality of the bacteriophage preparation, particularly the elimination of endotoxins, influenced the extent of the immune response following pre-infection treatments (data not shown). This is in contrast to post-infection treatments, where the immune system has already been primed by bacteria when the bacteriophages were administered.

Humans are constantly exposed to bacterial viruses —an estimated 10^31^ are on the Earth [20]—but little is known about the immunological consequences of this. Recently, a metagenomic analysis of viral DNA present in the lungs of CF patients identified over 100 different viral genomes, and this was considered to be a low diversity compared with other environments [21]. Nevertheless, the role of the immune system in shaping viral diversity has yet to be deeply studied. Furthermore, the immune response to therapeutic bacteriophages has not been extensively investigated because most of the available data were obtained from model viruses which have little interest as therapeutic agents [22], [23], [24].

Bacteriophage treatments of *P. aeruginosa* chronic lung infections in animal models have still not been investigated, and no specific procedure has been developed to isolate bacteriophages with higher infectivity against chronic clinical strains. However, several lines of evidence are encouraging. First, some bacteriophages are known to possess hydrolases that degrade bacterial exopolysaccharides [13], [25]. Second, several *in vitro* biofilm models have shown that bacteriophages can access and infect bacteria grown under these conditions [26], [27]. Third, mucus overproduction could be controlled or reduced as recently shown using a cPLA2α inhibitor [28]. Fourth, our study suggests that the use of bacteriophages devoid of endotoxins should not induce a strong stimulation of the pro-inflammatory markers.

Finally, optimization of bacteriophages can extend their host ranges. In the present study, full sequencing of the optimized bacteriophage revealed that only two single nucleotide mutations are sufficient to improve its virulence towards a clinical strain. This indicates the advantages of rapid bacteriophage evolution over conventional drugs that can take months if not years to optimize.

Besides the present study, phage therapy on lung infection caused by *Burkholderia cenocepacia* has also been reported [17]. Although there are some dissimilarities, this might be due more to the pathogens than to the phage treatments as these two bacteria do not elicit an identical response in the host [29], [30]. These studies suggest that bacterial lung infections can be treated by bacteriophages as long as the bacteria remain accessible (phage therapy is not suitable for intracellular bacteria).

The preventive treatment appears to be of lesser interest as its clinical application might not be as wide as the curative treatment. However it should not be disregarded as its efficacy was impressive; a single dose retained efficacy for up to four days. The limited clinical application is due to the fact that it is difficult to know in advance which bacterial strains will infect an invidual. Nevertheless, some individuals—such as CF or immuno-compromized patients— are known to be more susceptible to hospital-acquired infections. These infections are sometimes due to identified pathogens, as in the case of epidemic strains [31]. As a consequence, as soon as an epidemic is identified, such a population of patients could benefit from preventive treatments to reduce the probability of becoming infected.

Bacteriophages should not be considered only as a stand-alone treatment. Their use in combination with already approved treatments (like antibiotics) will be most likely the best form of application. Experimental, clinical, and regulatory data of such combined treatments are now urgently needed since antibiotic resistance is still rising [5].

Together with our previous work, we have now demonstrated that two different bacteriophages administrated intranasally are effective in treating lung infections with two different bacterial strains of *P. aeruginosa*. This extends our knowledge concerning the use of bacterial viruses in the treatment of pulmonary infections.

## Materials and Methods

### Ethics Statement

Animals were housed in the Institut Pasteur animal facilities accredited by the French Ministry of Agriculture to perform experiments on live mice, in appliance of the French and European regulations on care and protection of the Laboratory Animals. Protocols were approved by the veterinary staff of the Institut Pasteur animal facility (approval ID 10.565).

### *Pseudomonas aeruginosa* strains

The CHA strain was cultivated at 37°C in LB medium with shaking and prepared as previously described [14]. The CHA strain is resistant to tetracyclin, chloramphenicol, ampicilin, streptomycin, nalidixic acid, spectinomycin, erythromycin and rifampicin and shows an intermediate level of resistance to gentamycin and ticarcillin [18], [19]. The 20 clinical CF strains were provided by P. Plésiat, Besançon, France.

### Bacteriophage isolation and preparation

Bacteriophage PAK-P3 was isolated from sewage water as previously described [14]. To adapt the PAK-P3 bacteriophage to the CHA strain, a liquid culture of the CHA strain was infected with PAK-P3 (MOI of 1/1000) and incubated at 37°C with shaking. After 4 hours, the culture, showing signs of lysis, was stopped by adding few drops of chloroform. The culture was then centrifuged at 8000 g for 10 min, and the supernatant was stored at 4°C before use in the next round of amplification. This was repeated five times and the final supernatant was diluted and plated onto a Petri dish overlaid with the CHA stain. A few individual plaques were picked and resuspended in SM buffer (10 mM Tris HCl pH7, 200 mM NaCl, 0.03% gelatine) and subsequently used for four cycles of plaque purification. A final set of 10 isolated plaques were chosen and tested for their host range against a set of 20 CF strains of *P. aeruginosa* by spotting serial dilutions of bacteriophages on bacterial lawns. Each of the 10 isolated plaques showed the same host range, and one was chosen and named bacteriophage P3-CHA. Liquid cultures of 1 litre were used for large-scale preparation of PAK-P3 and P3-CHA using caesium chloride ultracentrifugation as described by Boulanger [32]. Bacteriophage solutions obtained were diluted in PBS for the curative treatment. Before use for the preventive treatment bacteriophage solutions were passed five times through an endotoxin-removal column (EndoTrap blue, Hyglos, Germany). The endotoxin-free bacteriophage solution contained 1×10^−10^ endotoxin units per pfu which correspond to 0.02UE per mice for the highest dose of bacteriophage given (1×10^8^ pfu in 50 µl). This value is over 100 fold inferior to the limited value accepted in Europe for bacterial endotoxins contamination of products that are administrated intraveneously (5.0 UE/kg body mass/hour; European Pharmacopoeia 5.0, 2005). Endotoxin concentrations were determined using the QCL-1000® Chromogenic LAL endpoint assay (Cambrex, Walkersville, MD, USA).

Heat-inactivated bacteriophage solution was obtained by a 10 min incubation at 80°C. Inability of heated bacteriophage to form plaque on bacterial lawns was checked.

Electron microscopy observations were performed on a Jeol 1200 EXII microscope after uranyl acetate staining of caesium chloride bacteriophage preparations [33].

Serial dilutions of bacteriophages PAK-P3 and P3-CHA were spotted on bacterial lawns for each of the 20 clinical strains. Efficacy of plating was calculated by comparison with an efficacy of 100% for respectively the PAK-P3 bacteriophage on the PAK strain and the P3-CHA bacteriophage on the CHA strain.

### Genomic characterisation of bacteriophages

Genome sequencing (20 to 25× coverage) was performed by Eurofins and Beckman Coulter Genomics using 454 technology with DNA prepared by standard procedures. The complete genome sequences of the PAK-P3 (vB_PaeM_PAK_P3) and P3-CHA (vB_PaeM_P3_CHA) bacteriophages are accessible in Genbank (accession numbers HM173082 and HM173081, respectively). Sequence analysis to search for temperate bacteriophage markers (similar genes) was performed on the PAK-P3 genome as described previously [14]. PAK-P3 and P3-CHA genomes were compared to search for mutations using Blast2seq tool. Two mutations were found in putative orfs 13 and 151 (nucleotide 7393 changed from A to G and nucleotide 69922 changed from G to A) leading to amino-acid substitutions Q to R and E to K, respectively in these two hypothetical proteins.

Total bacteriophage proteins were run on an SDS gel and subjected to in-gel trypsin digestion; microsequencing of the major capsid proteins was then performed by the Institut Pasteur microsequencing facility. None of the peptide sequences corresponded to any protein sequences in the databank before inclusion of our bacteriophage sequence data.

### Animal Infection

Mice (8 weeks old Balb/c males) were supplied by the Centre d'élevage R. Janvier. Food and drink were provided ad libitum. Animal infections and treatments were performed as described previously [14].

Bronchoalveolar lavages (BAL; 4×0.5 ml) were performed 20 h after infection following pentobarbital euthanasia (300 mg/kg). One aliquot of the BAL fluids was centrifuged for 10 minutes at 1400 rpm and murine cytokine concentrations were determined using DuoSet ELISA kits from R&D Systems. LDH activity was estimated using the Cytotox96 kit from Promega. The release of LDH in extracellular media reflects organ toxicity. Another aliquot of the BAL fluids was centrifuged at 6000 rpm for 10 minutes to separate free bacteriophages from bacteria. To determine the amount of free bacteriophages, supernatants were diluted and spotted onto plates overlaid with the CHA strain. To determine viable bacterial counts, pellets were resuspended in PBS, serially diluted and plated onto LB agar plates.

### Histological and immuno-histochemistry analyses

Lungs were removed from euthanized animals, fixed in 4% buffered formaline for at least 24 h at 4°C, and then embedded in paraffin. Serial 4 µm-thick sections were stained with haematoxylin-eosin (HE) and Gram. Histological scoring of elementary lesions of alveolitis, bronchitis, necrosis, and consolidation were scored by an investigator, who was blind to the treatment group, from 0 to 5 (0: none, 1: mild, 2: moderate focal, 3: moderate focal and diffuse, 4: severe focal, 5: severe focal and diffuse extending to the whole lobule). Recruitment of lymphocytes, alveolar macrophages - monocytes and polymorphonuclear cells was similarly evaluated from 0 to 3 (0: no, 1: weak, 2: moderate and 3: severe). Addition of these individual scores gave a lesion-severity score ranging from 0 to 25. Immunohistochemistry of infected lungs was performed with a primary polyclonal anti-*Pseudomonas* antibody raised in rabbit against the PcrV protein [34]. Affinity-purified antibodies were diluted 1/4000 before use. The method used was a classical Envision Dako protocol performed with amino-ethyl–carbazol as a chromogen.

### Statistical analysis

Mantel-Cox tests were performed for survival curves and one-tailed Mann-Whitney paired tests were used to compare quantifications of bacteria, bacteriophages and cytokines. Both were calculated with Prism 4.0 software (France). Error bars are s.e.m.
